# Heritability and Genetic Advance among Chili Pepper Genotypes for Heat Tolerance and Morphophysiological Characteristics

**DOI:** 10.1155/2014/308042

**Published:** 2014-11-16

**Authors:** Magaji G. Usman, M. Y. Rafii, M. R. Ismail, M. A. Malek, Mohammad Abdul Latif

**Affiliations:** ^1^Department of Crop Science, Faculty of Agriculture, Universiti Putra Malaysia (UPM), 43400 Serdang, Selangor, Malaysia; ^2^Institute of Tropical Agriculture, Universiti Putra Malaysia (UPM), 43400 Serdang, Selangor, Malaysia; ^3^Bangladesh Institute of Nuclear Agriculture, Mymensingh 2202, Bangladesh; ^4^Bangladesh Rice Research Institute, Gazipur 1701, Bangladesh

## Abstract

High temperature tolerance is an important component of adaptation to arid and semiarid cropping environment in chili pepper. Two experiments were carried out to study the genetic variability among chili pepper for heat tolerance and morphophysiological traits and to estimate heritability and genetic advance expected from selection. There was a highly significant variation among the genotypes in response to high temperature (CMT), photosynthesis rate, plant height, disease incidence, fruit length, fruit weight, number of fruits, and yield per plant. At 5% selection intensity, high genetic advance as percent of the mean (>20%) was observed for CMT, photosynthesis rate, fruit length, fruit weight, number of fruits, and yield per plant. Similarly, high heritability (>60%) was also observed indicating the substantial effect of additive gene more than the environmental effect. Yield per plant showed strong to moderately positive correlations (*r* = 0.23–0.56) at phenotypic level while at genotypic level correlation coefficient ranged from 0.16 to 0.72 for CMT, plant height, fruit length, and number of fruits. Cluster analysis revealed eight groups and Group VIII recorded the highest CMT and yield. Group IV recorded 13 genotypes while Groups II, VII, and VIII recorded one each. The results showed that the availability of genetic variance could be useful for exploitation through selection for further breeding purposes.

## 1. Introduction

Chili pepper (*Capsicum annuum* L. and* Capsicum frutescence* L.) is widely cultivated, primarily as a spice crop [1]. The optimum day temperatures for chili pepper growth range from 20 to 30°C [2], and day temperatures rise above 30°C year round in Malaysia [3]. The domestic production of chili pepper in Malaysia can hardly meet 70% of demand due to poor performance of local varieties under high temperature. Furthermore, high temperature is one of the major problems for chili cultivation in Malaysia. Such conditions are the important factors limiting the production of chili pepper. Therefore, understanding the effect and mechanism of high temperature on chili pepper are the important factors for the improvement of the quality of the crop. However, chili pepper, as well as other crops such as groundnut [4] and heat tolerant genotypes will be needed to sustain their production under high temperature environments.

Yield is a determining factor for crop improvement [5]. Chili pepper, as in other crops, yield is a quantitative trait that is influenced by a number of yield contributing parameters. The selection of desirable genotypes is usually based on yield and yield components. It is therefore necessary to study the mutual relationship between yield and yield components for efficient utilization of the genetic stock in crop improvement program of chili pepper. Variability in plants is the first step in understanding how to improve or produce new plants. Heritability is the degree of genetic control associated to some important traits [6]. It indicates how much of the genetic variability has a genetic origin and gives necessary information for the genetic selection process [7]. To improve grain yield potentials of crops in any breeding programs, it is necessary to obtain adequate information on the magnitude and type of genetic variability and their corresponding heritability. This is because selection of superior genotypes is proportional to the amount of genetic variability present and the extent to which the characters are inherited. Heritability is used to indicate the relative degree to which a character is transmitted from parent to offspring. The magnitude of such estimates also suggests the extent to which improvement is possible through selection [5].

Temperature and other abiotic stresses are clearly limiting factors for the growth and development of crop. Indeed stresses due to high temperature can be harmful to all phases of plant development, and global climate change is thought to cause extreme environmental fluctuations in most agricultural regions [8]. Temperature increment due to changing climatic condition is a serious threat [9] which affects crop production. So, understanding how the plants respond to stress is a challenging area of research [10]. Cell membrane thermostability (CMT) is a phenotypic parameter used in measuring electrolyte leakage from leafs of plants at different temperatures. CMT is a very sensitive and rapid method to identify heat tolerance in plants [11]. Several studies have indicated that CMT is effective in detecting genetic difference among several crops for heat tolerance [12, 13]. However, the mechanism for heat tolerance using the electrolyte leakage of crops under heat stress need to be more exploited in order to identify heat tolerant lines for the development of high yielding heat tolerant hybrid varieties, which will contribute to achieve self-sufficiency in chili production in Malaysia. Genotypes within several crop species are found to differ with respect to heat tolerance, where heat tolerant genotypes are referred to as giving the highest yield under high temperature condition [14–16]. Heat tolerance can be referred to as performance of a plant with respect to its yield or physiological processes under elevated temperature as compared with its performance under optimal temperature [17]. Genotypic differences in tolerance/susceptibility have been reported in chili pepper for pollen tube length and pollen germination and membrane stability [18, 19]. However, only a very limited number of genotypes of chili pepper have being studied. Furthermore, association between heat tolerance and cell membrane thermostability with respect to selection criteria for breeding purposes has not been investigated.

The objectives of this research was to identify chili pepper genotypes tolerant to high temperature and study the genotypic variation among the genotypes for heat tolerance and other yield related traits, to determine whether genotypic differences in tolerance to high temperature were associated with membrane thermostability, to study the correlation between heat tolerance and morpho-physiological traits and determine whether heat tolerance can be used as selection criteria in chili pepper breeding program.

## 2. Materials and Methods

### 2.1. Site and Location

This research was conducted between May and August 2013 using the Rain Shelter facility of the Agro-technology experimental site, Institute of Tropical Agriculture, Universiti Putra Malaysia. It is located on 3°02′ N latitude and 101°42′ East longitude and altitude is 31 m above sea level. The average climatic conditions are represented in Table 1.

### 2.2. Plant Husbandry

Experiment was carried out in a prepared polythene bag under rain shelter condition. All genotypes used for this experiment were collected from Asian Vegetable Research and Development Centre, AVRDC, in Taiwan, except for Kulai, which is a local variety cultivated in Malaysia (Table 2). Thirty-six genotypes belonging to two species of* Capsicum* were sown in seed trays on 8th May, 2013, with 1-2 seeds per cell and the growing medium was peat moss. They were later transplanted to the pots filled with cocoa peat. The pots were 17 cm × 30 cm with small holes to drain excess water. The experiment was laid in a randomized complete block design (RCBD) in three replications. Two pots were assigned for each genotype in each replication (72 pots per replication). The pots were oriented east to west and spaced 75 cm × 150 cm. Seedling emerged within 7–10 days after sowing and were transplanted 4 weeks after sowing. All pots were irrigated and fertilized using fertigation system of cropping with drip system of irrigation.

### 2.3. Procedure for Cell Membrane Thermostability

The membrane stability test was conducted at the Plant Physiology laboratory, Department of Crop Science, Universiti Putra Malaysia. The leaf cell membrane thermostability (CMT) in the chili pepper genotypes was assessed according to the procedure described [20]. A sample for assay consists of a paired set, namely, control (*C*) and treatment (*T*) set of 6 leaf disks each 1.3 cm^2^. The disks were cut from five fully expanded 3rd or 4th leaves from the top of the stem axis from each genotype. Samples were replicated three times each. Prior to assay, the paired set of leaf disks were placed in two separate test tubes (50 mL) and washed thoroughly with four exchanges of de-ionized water, 10 mL each time, to remove electrolytes adhering to the cut surface of the leaf discs. After the final wash, both sets of test tubes were filled with 10 mL de-ionized water and sealed with aluminium foil to avoid evaporation. The *T* set of the test tubes were incubated for 20 min at 50°C in a temperature controlled water bath, while the *C* set of test tubes were kept at room temperature (approximately 25°C). Both sets of test tubes were then incubated at 4°C (kept in a refrigerator) for 24 hrs. Initial conductance reading of both sets (CEC 1 and TEC 1) was made using an electrical conductivity meter (Starter) after bringing test tubes to room temperature. Tubes were then sealed again with aluminium foil and autoclaved at 121°C and 0.15 MPa for 20 min. to completely kill the leaf tissue. Autoclaved tubes were cooled to room temperature; content was mixed thoroughly and final conductance (CEC 2 and TEC 2) reading was taken. The CMT was calculated using the following equation, where TEC and CEC are a measure of conductance in treated and control test tubes, respectively, at initial (CEC 1 and TEC 1) and final (CEC 2 and TEC 2) conductance measurements [19]:

(1) $$\begin{array}{c} \text{CMT}\left(\%\right)=\frac{1-\left(\text{TEC}\;1/\text{TEC}\;2\right)}{1-\left(\text{CEC}\;1/\text{CEC}\;2\right)}\times 100. \end{array}$$

### 2.4. Morpho-Physiological Parameters

Characters assessed include disease incidence (%), plant height (cm), days to flowering, fruit length (cm), fruit weight (g), number of fruits, yield per plant (g), chlorophyll content, photosynthesis rate, and degree of pungency.

### 2.5. Chlorophyll Determination

Measurement of chlorophyll content was done following [21] procedure. Fresh leaves were collected from each replication from each genotype. One cm^2^ leaf disks were cut from the leaves using leaf puncher and were transferred immediately into scintillation vials containing 20 mL of 80% acetone. The vials were capped and covered with aluminium foil after which kept in the dark for 7 days. UV spectrophotometer was used to measure the absorbance at 647 and 664 nm wavelengths. Using the following formulas, total actual chlorophyll was calculated:

(2) $$\begin{array}{c} \text{Chlorophyll}\;\text{a}=\left(13.19\times A_{664}\right)-\left(2.57\times A_{647}\right),\\ \text{Chlorophyll}\;\text{b}=\left(22.1\times A_{647}\right)-\left(5.26\times A_{664}\right),\\ \text{Total}\;\text{chlorophyll}=\text{Chlorophyll}\;\text{a}+\text{chlorophyll}\;\text{b},\\ \text{Actual}\;\text{chlorophyll}=\frac{\left(3.5\times \text{total}\;\text{chlorophyll}\right)}{1}. \end{array}$$

### 2.6. Photosynthesis Rate (*µ*mol/m^2^/s) Measurement

Net photosynthetic for the 36 genotypes was measured from the leaves of 90-day-old seedlings. The uppermost expanded leaves were selected for the measurement using an LI-6400XT portable photosynthesis system (LiCOR Inc., Lincoln, Nebraska, USA).

### 2.7. Degree of Pungency

The extractions of capsaicin from the chili pepper samples was done using the method described [22] and were analyzed using Ultra Fast Liquid Chromatography (UFLC) as described [23]. Whole chili fruits from the 36 genotypes were collected for determining degree of pungency (spicy level). Scoville Heat Units was used to calculate the heat units for all samples. Scoville Heat Units are calculated in parts per million of heat (ppmH) based on sample dry weight according to the following formula from [24]:

(3) ppmH=Peak  area  of  capsaicin+0.82peak  area  of  dihydrocapsaicin×ppm  standardmL  acetonitrile×Total  capsaicin  peak  area  of  standardg  sample−1.

Conversion to Scoville Heat Units was made by multiplying ppmH by a factor of 15. They are classified as follows:

- (0–700 SHU) nonpungent
- (700–3,000 SHU) mildly pungent
- (25,000–70,000 SHU) highly pungent
- (3,000–25,000 SHU) moderately pungent
- (>80,000 SHU) very highly pungent [25].

### 2.8. Statistical Analysis

Results were analyzed using SAS software (version 9.1) for all traits and means were separated using Duncan's Multiple Range Test (DMRT) and Least Significance Difference (LSD) at 5% level.

#### 2.8.1. Phenotypic and Genotypic Coefficient of Variation

The estimates of phenotypic and genotypic coefficient of variation were calculated as described [26] as follows:

(4) $$\begin{array}{c} \text{PCV}\left(\%\right)=\frac{\sqrt{\text{Vp}}}{\text{mean}}\times 100,\;\;\text{GCV}\left(\%\right)=\frac{\sqrt{\text{Vg}}}{\text{mean}}\times 100, \end{array}$$

where PCV is phenotypic coefficient of Variance, VP is phenotypic variance, GCV is Genotypic Coefficient of Variance, and Vg is genotypic variance. GCV and PCV values were categorized as low (0–10%), moderate (10–20%), and high (20% and above) as indicated by [27, 28].

#### 2.8.2. Heritability

It was estimated as the ratio of total genotypic variance to the phenotypic variance according to [7]:

(5) $$\begin{array}{c} H^{2}=\frac{\text{Vg}}{\text{Vp}}\times 100, \end{array}$$

where *H* ^2^ = % Broad sense heritability. The heritability percentage was categorized as low (0–30%), moderate (30–60%), and high ≥60% as given by [29].

#### 2.8.3. Genetic Advance

The extent of genetic advance expected through selection for the character was calculated as in [29]:

(6) $$\begin{array}{c} \text{Genetic}\;\text{Advance}\;\left(\text{GA}\right):H\times P\times K, \end{array}$$

where *H* is heritability, *P* is phenotypic standard deviation, and *K* is selection deferential (2.06 at 5%).

Genetic Gain (%) = GA × 100; it is categorized as low (0–10%), moderate (10–20%) and high (20% and above) [29].

### 2.9. Multivariate Analysis

Cluster and Principal Component Analysis were carried out to assess the genetic diversity of cell membrane thermostability and yield characters using NTSYS-pc software (version 2.1). Data were analyzed based on Euclidian distance method, dissimilarity coefficient. In order to determine the genetic relationships among the chili pepper accessions the UPGMA algorithm and SAHN clustering were applied. The PCA of the 36 chili pepper genotypes were calculated by EIGEN module of NTSYS-pc software.

## 3. Results

### 3.1. Cell Membrane Thermostability (CMT)

Looking at the climatic condition at which the plants were grown, cell membrane thermostability would prove effective for screening the genotypes for heat tolerance. The result from this study showed that there was a highly significant (*P* < 0.01) difference among the genotypes in the relative injury (RI) and cell membrane thermostability (CMT) (Table 3). The cell membrane thermostability values ranged from 11.70 (AVPP9901) to 89.27% (AVPP0702) with a mean value of 64.62%. Most of the genotypes were recorded with high CMT values (>60%). Genotypes AVPP0105, AVPP9905, AVPP0116, AVPP0506, AVPP0803, C05573, AVPP0014, AVPP0702, AVPP0513, and AVPP0904 were found to be the most heat tolerant with the highest CMT values, while genotypes AVPP9703, AVPP9901, and AVPP0002 recorded the lowest CMT values indicating sensitivity to heat at 50°C.

### 3.2. Morphophysiological Characters

All the morphophysiological characters studied in this experiment showed highly significant (*P* < 0.01) difference except days to flowering and chlorophyll content (Table 4). The means for the 9 traits are presented in Table 5. The yield per plant ranged from 108.69 (AVPP0804) to 1144.3 g (AVPP9905). The plant height at harvest ranged from 41.4 to 92.13 cm which were recorded by AVPP0804 and AVPP0116 genotypes, respectively (Table 5). Significant range of variations was also observed for plant height at harvest, disease incidence, fruit length, fruit weight, and number of fruits. Similarly, a highly significant (*P* < 0.01) variation was observed among the 36 genotypes for photosynthesis rate and degree of pungency. The genotype Kulai recorded the highest rate of photosynthesis (19.32) while AVPP0705 gave the lowest (3.75). The average rate of photosynthesis in this investigation was to be 13.29 *µ*mol/m^2^/s. Degree of pungency or spicy level of the chili were found to vary significantly (*P* < 0.01) among the genotypes (Table 4). The genotype AVPP0705 showed the highest pungency (247245.28 SHU). The capsaicin and dihydrocapsaicin contents ranged from 175.26 to 133.1 mg/L, respectively (Table 6). However, no significant variation was observed for chlorophyll content and days to flowering.

### 3.3. Genotypic Variation for Heat Tolerance and Yield Characters in Chili Pepper

**Figure 1:**
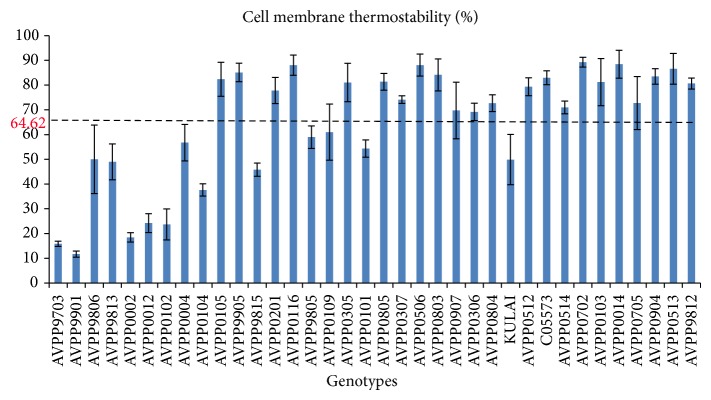
Variation among 36 chili pepper genotypes in cell membrane thermostability.

**Figure 2:**
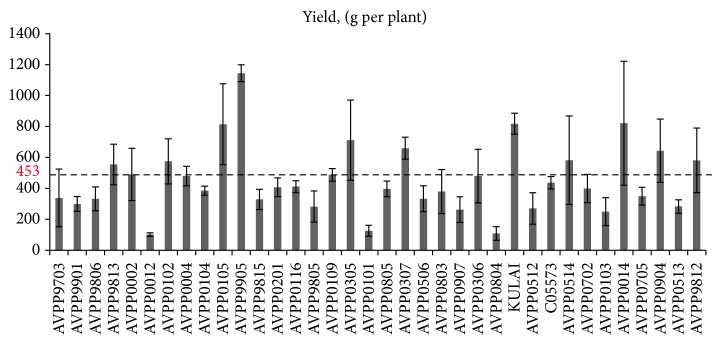
Variation in yield per plant for 36 genotypes of* Capsicum spp.*

The significant variation among the genotypes for heat tolerance is shown in Figure 1. The figure showed that the genotypes varied with respect to heat tolerance. Most of the genotypes were found to be above the mean.  Figure 2 showed variation in the genotypes with respect to yield per plant. Similarly, significant variations were observed for plant height, number of fruits, fruit length, disease incidence, and fruit weight.

### 3.4. Heritability and Genetic Advance as Indices for Heat Tolerance and Morpho-Physiological Characters Selection in Chili Pepper

#### 3.4.1. Coefficient of Variation

The extent of variability in respect of phenotypic and genotypic variances and phenotypic and genotypic coefficients of variance (GCV) for CMT and morphophysiological characters is represented in Table 6. The GCV estimate ranged from 1.73 (DF) to 75.78 (FW) over all the characters.

Moreover, high GCV and PCV were observed (>20%) in the traits for cell membrane thermostability, photosynthesis rate, disease incidence, fruit weight, and number of fruits and yield. Moderate GCV were also recorded for plant height and fruit length. The lowest were recorded for days to flowering and fruit length (<10%) (Table 7).

#### 3.4.2. Heritability and Genetic Advance

Broad sense heritability values for the ten traits are presented in Table 7. Traits such as membrane thermostability, photosynthesis rate, fruit weight, and fruit length showed a relatively high heritability values (>60%). The values estimated for plant height at harvest, number of fruits and yield per plant were moderate (30–60%). Fruit length, fruit weight, number of fruits, yield, and cell membrane thermostability exhibited the highest predicted genetic advance as compared to the other traits. High heritability (>60%) together with high genetic advance (>20%) was observed for CMT, photosynthesis rate, fruit weight, and fruit length traits. However, moderate heritability with high genetic advance was observed for number of fruits and yield per plant (Table 7).

#### 3.4.3. Correlation Coefficient

Simple phenotypic correlation coefficients for the traits studied using SAS software (version 9.1) are shown in Table 8. Yield per plant showed strong to moderately positive correlations (*r* = 0.23–0.56) at phenotypic level while at genotypic level correlation coefficient ranged from (0.16 to 0.72) for plant height at 6WAT and at harvest, days to flowering, fruit length, and number of fruits. However, negative correlation was observed for disease incidence with yield per plant. Similarly, cell membrane thermostability was found to correlate negatively with disease incidence. Positive phenotypic correlation between CMT and plant height and genotypic correlation between CMT and yield were also observed. Other correlation coefficients between pairs of traits that are of some interest to the breeders are shown in Table 8.

### 3.5. Genetic Distance-Based Analysis for Cell Membrane Thermostability and Yield

#### 3.5.1. Cluster Analysis

**Figure 3:**
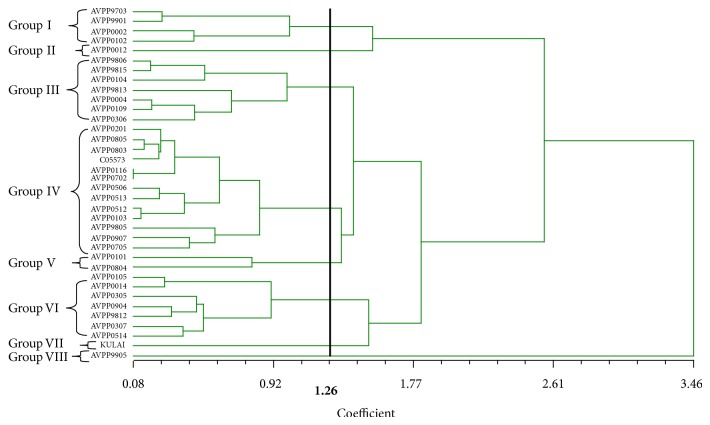
Relationship among the 36 chilli pepper genotypes based on cell membrane thermostability and yield characters using SAHN clustering on UPGMA method.

The standardized data was employed to calculate the Euclidean distances among the 36 chili pepper genotypes and an UPGMA dendrogram was constructed (Figure 3). The dendrograms of the 36 chili pepper genotypes were grouped into 8 major groups based on the cell membrane thermostability and yield traits at 1.26 dissimilarity coefficients. The cutoff at this point was for the convenience of discussion. Group IV recorded the highest number with 13 genotypes followed by groups III and VI with 7 genotypes each. Group I and V recorded 4 and 2 genotypes, respectively, while groups II, VII, and VIII had 1 genotype each. Group VIII gave the highest CMT value and recorded the highest yield, while Group II gave the lowest yield and Group I gave the lowest CMT value (Table 9).

#### 3.5.2. Principal Component Analysis

**Figure 4:**
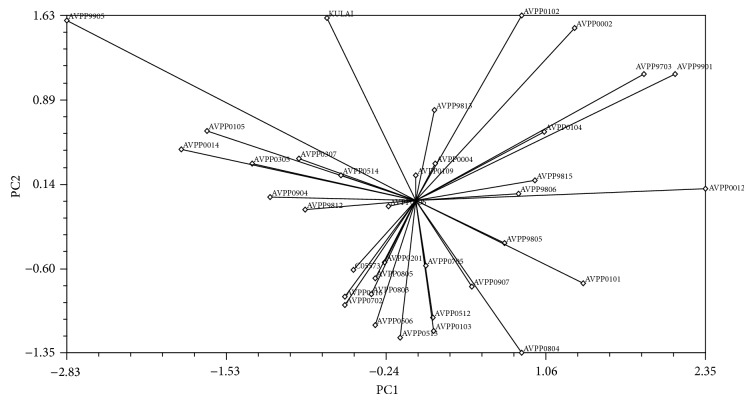
Two-dimensional graph principal component analysis showing relationship among the genotypes for cell membrane thermostability and yield.

Principal component analysis was carried out on the cell membrane thermostability and yield per plant characters on the 36 genotypes. The two-dimensional graphical illustration (Figure 4) showed that most of the genotypes were dispersed at close distance while few were dispersed at great distances as reflected by the Eigen vector. The farthest genotypes from the centroid were AVPP9905, Kulai, AVPP0102, AVPP0002, AVPP9901, AVPP9703, AVPP0012, AVPP0804, and AVPP0101, while other genotypes are more or less close to the centroid. The variation percentages of the PC1 and PC2 are 62.1 and 37.9%, respectively, with PC1 showing the highest of the total variation.

## 4. Discussion

### 4.1. Cell Membrane Thermostability (CMT)

The membrane is the first line of defense with many heat-responsive sensors that help plants to activate defense mechanisms early in heat shock. The mean value of CMT from this investigation was 64.62% indicating in most of the genotypes that membrane integrity was not damaged by the high temperature treatment of 50°C for 20 min. This shows that most of the genotypes assessed are tolerant to heat having relative injury of <40%. Increased electrolyte leakage indicates decreased cell membrane thermostability (CMT), which has long been used as an indirect mechanism of heat-stress tolerance in several crop plant species, including tomato, potato [30], and wheat [31]. Therefore membrane leakage can be used as a means of screening vegetative plants for heat tolerance in chili pepper. This is in conformity with the works of [19] that measured CMT in 12 ornamental pepper genotypes and found most of the genotypes to be thermotolerant with a mean of 59.50%. Membrane thermostability was also measured by [4] in diverse groundnut genotypes and observed heat tolerant genotypes has low percentage of membrane leakage, with high CMT. He further reported that genotypes of diverse origin indicate different sources of tolerance to heat.

### 4.2. Morphophysiological Characters

The investigation revealed considerable amount of variation for the characters studied. Such wide variation indicated the scope for improving the genotypes studied for these characters with respect to heat tolerance and morphophysiological characters. High performance chili pepper genotypes, namely, AVPP9905, AVPP0105, and Kulai, in terms of growth and yield were observed in this investigation. Photosynthesis rate is the rate at which the plant can photosynthesize its own food. It measures how efficient the plant is in carbon assimilation. The variation observed in this investigation among the 36 genotypes gave room for possible selection and improvement. Similar results were observed by [32, 33] who reported significant variation in growth and yield characters of pepper genotypes. On the contrary, [34] reported significant variation among the genotypes studied for days to 50% flowering. This might be attributed to the differences in the genetic sources of the genotypes studied. In the case of degree of pungency, genotype AVPP0705 was found to record the highest capsaicin content and was the highest in pungency which was significantly (*P* < 0.05) higher than all the genotypes tested. Genotypes AVPP9703, AVPP0512, AVPP0307, AVPP0803, and AVPP0102 were found to record no capsaicin and therefore be non-pungent. Similar variation in capsaicin content of different peppers has been previously reported [35–37]. In addition, capsaicin and dihydrocapsaicin have the same trend in contents of the capsaicinoids, and in particular capsaicin was found in higher contents than dihydrocapsaicin in all genotypes studied except C05573, AVPP9905, and AVPP9805.

### 4.3. Genetic Variation for Heat Tolerance and Yield Traits in Chili Pepper

The most tolerant genotypes seem to have high membrane stability (CMT > 60%) at high temperature of 50°C. Cell membrane thermostability therefore showed some potential for screening for heat tolerance in chili pepper. This is in line with the works of [38] who found large plant-to-plant variation in cell membrane thermostability measurements in Chrysanthemum cultivars. Most of the genotypes studied by [4] showed that membrane integrity was damaged by the high temperature treatment of 54°C for 15 min. On the contrary, [39] found no significant variation of CMT for ten lines of cabbage (*Brassica oleracea* L.). Heat tolerant genotypes having greater CMT at high temperature are said to have higher optimum and maximum temperatures for growth and development processes. This also indicates the effectiveness of cell membrane thermostability parameter as rapid and sensitive in determining heat tolerance among chili pepper genotypes. Similarly, variations were observed among the genotypes for yield indicating differences in their yield performance under the same condition. This might be due to the differences in their genetic make-up. This investigation is in harmony with the work of [32].

### 4.4. Heritability and Genetic Advance as Indices for Heat Tolerance and Morphophysiological Characters Selection in Chili Pepper

#### 4.4.1. Coefficient of Variation

The extent of genetic variation in heat tolerance as well as other morphophysiological characters is better judged by the estimation of the genotypic coefficient of variation (GCV) in relation to its phenotypic coefficient of variation (PCV). A small difference between GCV and PCV was observed in plant height at harvest, fruit length, fruit weight, number of fruits, and heat tolerance characters indicating that variations among the genotypes were mostly due to genetic factors. This indicates a high significant effect of genotype on phenotypic expression with very little effect of environment. On the other hand, large differences between GCV and PCV were observed for the characters days to flowering, disease incidence, chlorophyll content, and yield per plant. This indicates the influence of the environment over these characters. High GCV observed for some traits such as CMT and yield per plant in this investigation indicates their high variability and that further selection could be used to improve the genotypes for the traits, while low GCV indicates limited improvement for the traits through selection. This is similar to the works of [40].

#### 4.4.2. Heritability and Genetic Advance

Effective selection can be achieved only when additive effects are sufficiently higher than the environmental effect. Report from [29] showed that effectiveness of selection depends not only on heritability but also on genetic advance. High GCV together with high heritability and genetic advance provide more information than other parameters alone [41]. High heritability together with high genetic advance observed in this investigation indicates that these traits such as CMT are mainly controlled by additive type of genes. Therefore, selection may be effective for improving these traits in chili pepper. Works of [42] observed high heritability with genetic advance in several characters in chili. This investigation is in agreement with the work of [6] who observed high genetic advance accompanied with high heritability in drought tolerance in sorghum. In this study, cell membrane thermostability, fruit length, and fruit weight are found to be important characters to be taken into consideration for effective selection in pepper. Thus, selection in this population of chili pepper would prove successful once the fixed genetic component is freed from environmental influence.

#### 4.4.3. Correlation between Traits

Relationship existing between cell membrane thermostability and disease incidence indicating the tendency of heat tolerant genotypes possesses disease resistant traits as compared to heat sensitive genotypes. As revealed from this investigation heat tolerant genotypes may be disease resistant genotypes. The likeliness of negative correlation between cell membrane thermostability and disease score as found in this study has also been reported by [43]. On the other hand, no significant association between cell membrane thermostability (heat tolerant trait) and yield per plant was observed.

### 4.5. Genetic Distance-Based Analysis for Cell Membrane Thermostability and Yield

Genetic divergence is one of the criteria of parent selection. Knowledge of genetic diversity among plant populations and its quantitative assessment usually helps a breeder in choosing desirable parents for breeding program as selection of parents on the basis of divergence analysis would be more effective [44].

#### 4.5.1. Clustering Analysis

The 36 genotypes of* Capsicum spp* grouped into 8 clusters based on cell membrane thermostability and yield traits at distant coefficient of 1.26 indicates a level of diversity among the genotypes. Group VIII, which has only one genotype (AVPP9905), recorded the highest yield and CMT values and this proved that this genotype might have dissimilar genes as compared to the other genotypes for controlling these traits. To improve the heat tolerance of Kulai (an adaptable and susceptible genotype) that recorded the second highest yield it needs to be crossed with AVPP9905. Similarly, AVPP9905 can be crossed with AVPP0012 for high yield and heat tolerance. Similar results were obtained by [45] who studied significant variation in terms of morphological traits in distribution of chili pepper. Genetic diversity among 45 chili pepper genotypes studied by [46] observed six clusters and [16] reported eight distinct grouped in 50 accessions of chili.

#### 4.5.2. Principal Component Analysis

The principal component analysis (PCA) yielded eigenvalues of each principal component axis of ordination of genotypes with the first axis totally accounting for the variation among the genotypes. The result of the PCA further justifies the clustering analysis. Genetically similar genotypes were grouped together. According to [7], genetically distant parents are able to exert high heterosis. Considering the variation and diversity analysis of the genotypes genotype AVPP9905 having high yield and more tolerant to heat (high CMT) from group VIII, 13 genotypes from group IV with high CMT but low yield and 7 genotypes from group VI with high CMT and Kulai with high yield and low CMT from cluster VII were found promising. Therefore, for hybridization program crosses among these genotypes for heat tolerance and yield could be effective. Similar results were observed by [46, 47].

## 5. Conclusion

Cell membrane thermostability is a useful parameter for thermotolerance in chili pepper. The present data indicated that most of the genotypes studied are consistently tolerant to high temperature measured based on membrane thermostability phenotyping parameter. Furthermore, these genotypes are from diverse origin indicating different sources of heat tolerance. However, the genetic parameters discussed above are function of environmental variability, so estimates may differ in other environments. High and moderate heritability and high genetic advance shown by the different characters, namely, plant height at harvest, fruit length, fruit weight and number of fruits, yield, and cell membrane thermostability determine the genetic effects of the phenotypic expression of these characters that they are fundamentally from the additive type. The prevalence of genetic variance for heat tolerance and morphophysiological traits studied can be exploited through selection as the estimate of high broad sense heritability and genetic advance allows doing so.

Considering the group distance, agronomic performance, and variability the crosses between AVPP9905 and Kulai; Kulai and AVPP0105, AVPP0014, AVPP0305, AVPP0904, AVPP9812, AVPP0307, AVPP0514; Kulai and AVPP0201, AVPP0805, AVPP0803, C05573, AVPP0116, AVPP0702, AVPP0506, AVPP0513, AVPP0512, AVPP0103, AVPP9805, AVPP0907, and AVPP0705 could be suggested for future hybridization program for heat tolerance and high yield.

## Figures and Tables

**Table 1:** The average temperature, solar radiation, and rainfall of the experimental site during the period of the experiment (2013/2014).

Months	Temperature (°C)		Solar radiation MJ/m²	Rainfall (mm)
	Maximum	Minimum		
May	38.5	24.5	22.70	—
June	36.3	24.2	21.2	4.2
July	36.3	23.8	20.20	100
August	39.3	23.8	—	—

Source: Malaysian Meteorological Department (http://www.accuweather.com/en/my/kuala-lumpur/233776/month/233776?monyr=7/01/2013#), 2013.

**Table 2:** List of chili pepper genotypes and their pedigree, specie, and heat tolerance used in this experiment.

Genotype	Source	Specie	Pedigree	Heat tolerance^a^
AVPP9703	CCA 1410A	*C. annuum *	HDA210/Szechwan10/MC4	excellent
AVPP9901	CCA 3106	*C. annuum *	HM-19/155-42//G-4	good
AVPP9806	CCA 196-A	*C. annuum *	SlamChiliF1/HDA248	—^b^
AVPP9813	CCA 323	*C. Frutescens *	Kulim/HDA295 (Berke's Joy)	excellent
AVPP0002	CCA 3331	*C. annuum *	Arunalu/Tumpang	excellent
AVPP0012	Jin's Joy	*C. annuum *	Jin's Joy	fair
AVPP0102	CCA 3636	*C. annuum *	IR/LongFruit	excellent
AVPP0004	Maor/Perennial	*C. frutescens *	Maor/Perennial	good
AVPP0104	CCA 3468	*C. annuum *	MI-2/Taiwan83-168-1-1//MI-2	good
AVPP0105	CCA 321	*C. annuum *	Kulim/HDA248	excellent
AVPP9905	Susan's Joy	*C. annuum *	Susan's Joy	excellent
AVPP9815	CCA226A-3	*C. frutescens *	Corbadzijiski/HDA248	—
AVPP0201	CCA 4283	*C. annuum *	(Szechwan9/PerennialHDV)/Jin's Curlicue//F1 Hy Hot 3 F3 sel.	—
AVPP0116	CCA 3743	*C. annuum *	PSP-11/Jin's Delight//Kulai	excellent
AVPP9805	CCA 2345	*C. annuum *	HDA248/5^*^LongFruitA	
AVPP0109	MSH-1 seln	*C. annuum *	F1 MSH-1	excellent
AVPP0305	CCA 4824	*C. frutescens *	PBC325/PBC308	
AVPP0101	CCA 3617	*C. annuum *	FriesdorferSelex/LongFruit	excellent
AVPP0805	CCA6306-1-3-3-3-1	*C. annuum *	Jatilaba/CCA321//Jatilaba	—
AVPP0307	CCA 4860	*C. annuum *	LongThick/9852-173	—
AVPP0506	CCA5213	*C. frutescens *	PBC 481 sel.//Kulium/HDA248	—
AVPP0803	CCA6875-1-1-3-1-3	*C. annuum *	Var F/UF8752-3	—
AVPP0907		*C. annuum *	Kulim/HDA248//Lorai	—
AVPP0306	CCA 4851	*C. annuum *	VC232/9945-1856	—
AVPP0804	CCA6875-8-1-3-1-1	*C. annuum *	Var F/UF8752-3	—
AVPP0512	CCA5217	*C. frutescens *	Jin's Joy//Kulium/HDA248	—
AVPP0514	CCA5218	*C. annuum *	Jin's Joy//Kulai^*^3/PBC932	—
AVPP0702	CCA6023-9-1-1-2-1	*C. annuum *	Jin's Joy/4/Bangchang-selex///HDA210/Szechwan10//MC4	—
AVPP0103	CCA 260	*C. annuum *	Szechwan9/Perennial	excellent
AVPP0014	Mr. Lee No.3 seln	*C. annuum *	Mr. Lee No.3 selex	fair
AVPP0705	CCA5684-05-1-2-1-1	*C. frutescens *	SSK-1/0209-4//SSK-1/PBC495	—
AVPP0904		*C. annuum *	Jin's Joy//Kulim/HDA248/Jin's Joy//Kulim/HDA248	—
AVPP0513	CCA5218	*C. frutescens *	Jin's Joy//Kulai^*^3/PBC932	—
AVPP9812	CCA 336-B	*C. frutescens *	PBC385/HDA248	fair
C05573	PBC 142	*C. annuum *	Pant C-1 (long term ck.)	good
Kulai	Local	*C. annuum *		—

^a^AVRDC, unpublished data.

^b^Data not available.

**Table 3:** Relative injury as determined by cell membrane thermostability test at 25 and 50°C for 20 min for 36 genotypes of *Capsicum spp*.

GENOTYPE	Relative injury (%)			CMT (%)^v^
	25°C^a^	50°C^a^	Injury^*^	
AVPP9703	31.00	89.00	84.13	15.87
AVPP9901	26.33	91.2	88.30	11.70
AVPP9806	18.57	59.07	50.00	50.00
AVPP9813	22.00	62.10	51.03	48.97
AVPP0002	28.67	86.83	81.57	18.43
AVPP0012	22.07	81.23	75.77	24.23
AVPP0102	31.70	84.10	76.33	23.67
AVPP0004	23.53	56.93	43.27	56.73
AVPP0104	23.40	71.30	62.40	37.60
AVPP0105	25.23	38.57	17.67	82.33
AVPP9905	29.63	40.20	14.90	85.10
AVPP9815	30.93	68.43	54.20	45.80
AVPP0201	32.83	47.97	22.20	77.80
AVPP0116	32.67	40.90	11.97	88.0
AVPP9805	27.07	56.90	41.03	58.97
AVPP0109	36.03	61.40	39.03	60.97
AVPP0305	31.93	45.23	18.97	81.03
AVPP0101	42.47	68.50	45.63	54.37
AVPP0805	21.67	36.40	18.67	81.33
AVPP0307	23.20	43.03	25.90	74.10
AVPP0506	34.63	42.23	11.90	88.10
AVPP0803	21.50	34.20	15.87	84.13
AVPP0907	32.33	32.33	29.97	69.73
AVPP0306	26.27	49.07	30.83	69.17
AVPP0804	26.73	46.67	27.30	72.70
AVPP0512	23.40	39.27	20.67	79.33
AVPP0514	28.43	49.20	29.03	70.97
AVPP0702	33.20	40.43	10.73	89.27
AVPP0103	31.83	44.97	18.80	81.20
AVPP0014	27.20	35.40	11.57	88.43
AVPP0705	28.70	48.10	27.27	72.73
AVPP0904	27.23	39.20	16.50	83.50
AVPP0513	37.17	46.27	13.43	86.57
AVPP9812	29.80	43.37	19.37	80.63
C05573	25.97	38.47	17.03	82.97
Kulai	21.37	60.93	50.13	49.87

Means	28.24	53.92	35.37	64.62
LSD (*P* < 0.05)	7.39	11.95	17.43	17.39
SEM	0.61	1.67	2.37	2.37

Mean values were separated using Least Significance Difference at 5% level of probability.

^a^Calculated as (initial conductivity/final conductivity) × 100.

^*^Calculated as calibrated RI = {1 − [1 − (TEC1/TEC2)]/[1 − (CEC1/CEC2)]}  × 100.

^v^Calculated as [1 − (TEC1/TEC2)]/[1 − (CEC1/CEC2)] × 100.

LSD = Least significance difference.

SEM = Standard error of mean.

**Table 4:** Analysis of variance for 11 characters for the 36 *Capsicum spp*.

Traits	Genotype (DF = 35)	Error (DF = 70)	CV (%)
PLH6WK	200.00^*^	113.50	24.09
PLHH	478.23^**^	102.28	14.55
Days to flowering	19.90ns	16.24	6.31
Disease incidence	524.02^*^	318.52	49.07
Chlorophyll content	5.56ns	5.99	37.23
Photosynthesis rate	37.97^**^	0.0016	0.305
Fruit length	18.64^**^	2.73	14.26
Fruit weight	161.95^**^	3.84	20.45
Number of fruits	6456.66^**^	1385.09	46.42
Yield	146873^**^	50594.56	49.59
Membrane stability	1622.85^**^	112.73	16.43
SHUs	4945258353.7^**^	7448153.84	7.30

^*^Significant at 5%, ^**^highly significant at 1%, ns = not significant, PLHH: plant height at harvest, PLH6WK: plant height at 6 weeks after transplanting, SHUs = Scoville heat units.

**Table 5:** Means for 10 characters studied in 36 genotypes of *Capsicum spp*.

Genotype	PH6WK	PLHH	DF	DI (%)	CPL	Photo	FL (cm)	FW (g)	NF	*Y* (g/pl)
AVPP9703	45.07	60.60	63.00	31.25	5.64	14.28	10.79	9.08	49.33	338.08
AVPP9901	32.15	47.10	68.50	78.89	6.43	10.92	8.28	6.07	62.00	299.36
AVPP9806	41.25	77.87	60.67	31.59	6.30	15.24	13.41	10.93	47.00	332.37
AVPP9813	56.33	72.07	61.67	30.73	5.75	12.22	10.73	9.43	78.00	554.79
AVPP0002	37.62	60.23	62.83	40.79	6.30	10.92	9.09	3.29	208.33	490.44
AVPP0012	44.80	77.60	67.00	49.10	7.35	15.24	13.16	8.08	20.00	102.03
AVPP0102	56.73	74.23	61.75	39.53	9.65	12.22	12.37	10.30	61.67	574.83
AVPP0004	43.47	77.07	65.00	38.85	4.60	10.92	9.19	6.03	86.67	479.63
AVPP0104	40.85	59.13	60.50	9.11	7.01	15.24	8.72	2.34	150.33	385.10
AVPP0105	39.50	78.57	63.00	40.37	5.91	12.22	12.31	9.67	142.33	813.79
AVPP9905	43.97	68.27	62.67	41.24	5.57	12.74	16.43	22.94	63.00	1144.30
AVPP9815	27.43	50.93	62.33	36.71	7.43	14.58	9.76	6.03	61.67	329.39
AVPP0201	45.23	59.40	60.67	29.80	7.61	11.48	11.71	6.19	78.67	407.36
AVPP0116	60.75	92.13	64.50	28.14	5.60	17.62	10.55	11.65	46.33	411.35
AVPP9805	28.43	45.37	65.00	28.83	7.90	13.56	15.35	14.67	21.00	282.83
AVPP0109	40.07	66.67	63.83	41.86	7.07	16.93	11.29	4.27	130.33	487.72
AVPP0305	55.10	67.30	59.00	30.47	7.92	10.47	12.91	9.73	116.00	711.27
AVPP0101	54.20	65.03	64.50	61.19	7.00	15.71	9.67	8.69	27.33	126.65
AVPP0805	54.78	86.13	63.17	33.18	5.79	14.47	9.92	4.96	95.00	396.67
AVPP0307	49.30	88.67	65.50	26.91	11.93	11.69	14.45	10.71	71.33	659.66
AVPP0506	38.77	75.47	64.50	25.31	5.58	8.85	10.14	4.77	88.00	332.80
AVPP0803	33.70	61.90	64.00	22.82	5.81	16.04	15.45	44.13	8.00	379.74
AVPP0907	41.78	88.87	69.83	42.95	5.86	16.91	6.97	2.47	46.67	263.13
AVPP0306	45.43	71.90	66.00	55.85	6.11	11.13	11.33	9.69	81.67	479.70
AVPP0804	30.60	41.40	67.50	59.85	7.36	13.04	7.83	14.00	6.33	108.69
AVPP0512	49.08	55.07	61.33	41.27	7.29	13.12	12.86	9.66	61.33	270.53
AVPP0514	38.77	67.83	67.00	19.79	5.29	16.96	14.00	12.71	68.67	582.34
AVPP0702	39.13	56.47	63.67	39.14	6.72	10.04	12.66	8.61	73.00	398.99
AVPP0103	44.20	72.43	66.33	42.05	6.54	16.12	8.99	3.54	81.67	249.99
AVPP0014	48.32	68.07	62.33	29.74	6.00	13.09	13.83	10.79	113.00	821.47
AVPP0705	46.40	83.80	63.33	16.69	6.88	3.75	8.02	3.57	151.00	349.93
AVPP0904	55.62	79.97	65.00	38.00	4.54	7.60	12.33	8.13	106.33	643.22
AVPP0513	44.43	71.80	66.00	23.16	5.62	6.02	12.60	9.64	39.33	283.38
AVPP9812	44.50	76.00	62.00	29.05	5.87	13.07	11.57	7.01	100.67	581.27
C05573	42.88	85.53	67.25	35.22	6.04	18.41	10.87	3.51	180.33	436.92
Kulai	51.50	72.17	59.00	39.96	6.44	19.32	17.23	17.68	64.00	818.10

Mean	44.23	69.53	63.89	36.37	6.58	13.12	11.58	9.58	80.18	453.55
LSD (*P* < 0.05)	17.35	16.47	6.56	29.06	3.99	0.066	2.69	3.19	60.61	366.29
SEM	1.16	1.45	0.45	1.96	0.23	0.020	0.27	0.72	5.53	28.57

PLH6WK: Plant height at 6 weeks; PLHH: plant height at harvest; DF: days to flowering; DI: disease incidence; CPL: chlorophyll content; Photo: photosynthesis rate; FL: fruit length; FW: fruit weight; NF: number of fruits; *Y*: yield; LSD: least significance difference; SEM: standard error mean.

**Table 6:** The capsaicins and dihydrocapsaicin content of the thirty-six chili pepper genotype samples (mg/L).

Genotypes	Capsaicin (mg/L)	Dihydrocapsaicin (mg/L)	Scoville Heat Units	Pungency (SHUs' scale)
AVPP9703	0.00	0.00	0.00	Non-pungent
AVPP9901	205.16	84.17	36809.30	Highly pungent
AVPP9806	167.81	95.91	32731.96	Highly pungent
AVPP9813	23.28	0.00	3250.48	Moderately pungent
AVPP0002	476.03	405.55	105796.29	Very highly pungent
AVPP0012	97.87	50.52	18564.11	Moderately pungent
AVPP0102	0.00	0.00	0.00	Non-pungent
AVPP0004	13.61	0.00	1899.62	Mildly pungent
AVPP0104	439.32	445.64	104557.81	Very highly pungent
AVPP0105	120.34	78.47	24413.04	Moderately pungent
AVPP9905	175.43	241.83	47945.70	Highly pungent
AVPP9815	25.57	25.98	6090.68	Moderately pungent
AVPP0201	18.04	0.00	2519.57	Mildly pungent
AVPP0116	32.78	21.57	6669.59	Moderately pungent
AVPP9805	129.17	164.79	34015.68	Highly pungent
AVPP0109	57.15	55.10	13323.25	Moderately pungent
AVPP0305	313.05	360.08	78628.18	Highly pungent
AVPP0101	39.65	63.45	11688.78	Moderately pungent
AVPP0805	411.98	299.98	86615.90	Very highly pungent
AVPP0307	0.00	0.00	0.00	Non-pungent
AVPP0506	582.78	355.88	115887.85	Very highly pungent
AVPP0803	0.00	0.00	0.00	Non-pungent
AVPP0907	141.97	69.03	26517.41	Highly pungent
AVPP0306	107.97	84.69	23288.70	Moderately pungent
AVPP0804	18.099	0.00	2527.43	Mildly pungent
AVPP0512	0.00	0.00	0.00	Non-pungent
AVPP0514	216.78	137.03	43558.53	Highly pungent
AVPP0702	102.37	82.00	22245.67	Moderately pungent
AVPP0103	323.04	231.16	67524.10	Highly pungent
AVPP0014	48.28	13.99	8099.05	Moderately pungent
AVPP0705	1277.56	710.02	247245.28	Very highly pungent
AVPP0904	191.15	181.29	44270.75	Highly pungent
AVPP0513	70.21	51.25	15030.29	Moderately pungent
AVPP9812	23.06	12.31	4742.27	Moderately pungent
C05573	376.42	408.03	92127.13	Very highly pungent
Kulai	83.61	61.77	17665.58	Moderately pungent

Mean	175.26	133.10	37395.83	
LSD (*P* < 0.05)	19.67	36.88	5540.40	
SEM	7.22	12.31	5828.29	

LSD: Least significance difference, SEM: Standard error mean.

**Table 7:** Estimate of broad sense heritability, genotypic, and phenotypic coefficients of variance, relative differences between GCV and PCV, and genetic advance for ten traits in 36 *Capsicum spp*.

Traits	*H* ^2^ (%)	*σ* ^2^ *g* (%)	*σ* ^2^ *p* (%)	GCV (%)	PCV (%)	RD	GA (%)
PLH6WK	20.25	28.83	142.34	12.14	26.99	55.02	11.24
PLHH	55.06	125.32	227.59	16.1	21.7	25.81	17.11
DF	6.99	1.22	17.46	1.73	6.54	73.55	0.94
DI	17.7	68.50	387.02	22.76	54.09	57.92	19.72
CPL	2.56	−0.15	5.85	5.89	36.56	83.98	1.94
PHOTO	100	12.66	12.66	26.77	26.77	0	55
FL	66	5.30	8.03	19.88	24.47	18.76	33.27
FW	93.21	52.70	56.54	75.78	78.49	28.29	150.71
NF	54.96	1690.50	3075.60	51.28	69.17	25.86	78.31
*Y*	38.81	32092.80	82687.40	39.5	63.4	37.7	50.69
CMT	81.7	503.37	616.1	34.72	38.41	5.71	64.64

PLH6WK: plant height at 6 weeks; PLHH: plant height at harvest; DF: days to flowering; DI: disease incidence; CPL: chlorophyll content, PHOTO: photosynthesis rate; FL: fruit length; FW: fruit weight; NF: number of fruits; CMT: cell membrane thermostability; *H* ^2^: heritability; *σ* ^2^ *g*: genotypic variance; *σ* ^2^ *p*: phenotypic variance; *Y*: yield; PCV: phenotypic coefficients of variance; GCV: genotypic coefficients of variance; RD: relative difference between GCV and PCV; GA: genetic advance.

**Table 8:** Coefficient of phenotypic and genotypic (bold) correlations among the investigated traits in 36 *Capsicum spp*.

	PLHH	DF	DI	Photo	CPL	FL	FW	NF	*Y*	CMT
PLHH	1	**0.70** ^**^	−**0.49** ^**^	**0.01** ^**^	**NA**	**−0.03** ^**^	**−0.21** ^**^	**0.22ns**	**0.19** ^**^	**0.39ns**
DF	−0.11ns	1	**1** ^**^	** 1** ^**^	**NA**	**NA**	**−1** ^**^	**−0.23** ^*^	**−0.67** ^**^	**0.04ns**
DI	−0.21^*^	0.28^**^	1	**0.23** ^**^	**NA**	**−0.41** ^**^	**−0.19** ^**^	**−0.22** ^**^	**−0.21** ^**^	**−0.44** ^**^
Photo	0.01ns	0.04ns	0.08ns	1	**NA**	**1** ^**^	**NA**	**−0.02** ^**^	**NA**	**−0.25** ^**^
CPL	−0.04ns	−0.04ns	0.03ns	−0.04ns	1	**0.09** ^**^	**0.12** ^**^	**NA**	**NA**	**NA**
FL	0.03ns	−0.27^**^	−0.18ns	0.08ns	0.08ns	1	**0.67ns**	**−0.36** ^**^	**0.72** ^**^	**0.26** ^**^
FW	−0.14ns	−0.08ns	−0.04ns	0.12ns	−0.04ns	0.60^**^	1	**−0.59** ^**^	**0.31** ^**^	**0.20** ^**^
NF	0.27^**^	−0.27^**^	−0.26^**^	−0.06ns	−0.07ns	−0.13ns	−0.40^**^	1	**0.16** ^**^	**0.07ns**
*Y*	0.29^**^	−0.38^**^	−0.23^**^	−0.07ns	−0.07ns	0.39^**^	0.18ns	0.56^**^	1	**0.35** ^**^
CMT	0.20^*^	0.06ns	−0.24^**^	−0.22^**^	−0.16ns	0.13ns	0.17ns	−0.02ns	0.14ns	1

^*^Significant at 5%, ^**^highly significant at 1%, PLHH: plant height at harvest; DF: days to flowering; DI: disease incidence; CPL: chlorophyll content; FL: fruit length; FW: fruit weight; NF: number of fruits; CMT: cell membrane thermostability; Photo: Photosynthesis rate; ns: not significant; NA: not available; *Y*: yield.

**Table 9:** Mean values of cell membrane thermostability and yield per plant characters for 8 groups revealed by cluster analysis on 36 genotypes of *Capsicum spp*.

Clusters	Genotypes	Yield	CMT
Group I	AVPP9703, AVPP9901, AVPP0002, AVPP0102	425.68	17.42
Group II	AVPP0012	102.03	24.23
Group III	AVPP9806, AVPP9815, AVPP0104, AVPP9813, AVPP0004, AVPP0109, AVPP0306	435.53	52.75
Group IV	AVPP0201, AVPP0805, AVPP0803, C05573, AVPP0116, AVPP0702, AVPP0506, AVPP0513, AVPP0512, AVPP0103, AVPP9805, AVPP0907, AVPP0705	343.35	80.01
Group V	AVPP0101, AVPP0804	117.67	63.53
Group VI	AVPP0105, AVPP0014, AVPP0305, AVPP0904, AVPP9812, AVPP0307, AVPP0514	687.57	80.14
Group VII	Kulai	818.10	49.87
Group VIII	AVPP9905	1144.30	85.10

CMT: Cell membrane thermostability.
